# Staff perceptions of patient safety in the NHS ambulance services: an exploratory qualitative study

**DOI:** 10.29045/14784726.2022.03.6.4.18

**Published:** 2022-03-01

**Authors:** Keegan Shepard, Sally Spencer, Carol Kelly, Paresh Wankhade

**Affiliations:** University of Oxford ORCID iD: https://orcid.org/0000-0003-3867-9752; Edge Hill University; Edge Hill University; Edge Hill University

**Keywords:** NHS ambulance services, patient safety, perceptions

## Abstract

**Objectives:**

Most research investigating staff perceptions of patient safety has been based in primary care or hospitals, with little research on emergency services. Therefore, this study aimed to explore staff perceptions of patient safety in the NHS ambulance services.

**Design:**

A stratified qualitative study using semi-structured interviews.

**Setting:**

Three urban or rural ambulance service NHS trusts in England.

**Participants:**

A total of 44 participants from three organisational levels, including executives, managers and operational staff.

**Methods:**

The semi-structured interviews explored the interpretation and definition of patient safety, perceived risks, incident reporting, communication and organisational culture. The framework method of qualitative data analysis was used to analyse the interviews and NVivo software was used to manage and organise the data.

**Results:**

We identified five dominant themes: varied interpretation of patient safety; significant patient safety risks; reporting culture shift; communication; and organisational culture. The findings demonstrated that staff perceptions of patient safety ranged widely across the three organisational levels, while they remained consistent within those levels across the participating ambulance service NHS trusts in England.

**Conclusions:**

The findings suggest that participants from all organisational levels perceive that the NHS ambulance services have become much safer for patients over recent years, which signifies an awareness of the historical issues and how they have been addressed. The inclusion of three distinct ambulance service NHS trusts and organisational levels provides deepened insight into the perceptions of patient safety by staff. As the responses of participants were consistent across the three NHS trusts, the identified issues may be generic and have application in other ambulance and emergency service settings, with implications for health policy on a national basis.

## Introduction

While there have been many advancements in modern medicine, it remains clear that patients are still at great risk of danger during care. The focus on the concept of patient safety has steadily grown ever since the Institute of Medicine released reports in 1999 and 2001 which determined that approximately 44,000 to 98,000 patients die each year in the United States due to completely avoidable medical errors (Stelfox et al., 2006). Following these seminal reports, healthcare organisations began to establish a robust focus on patient safety; however, despite the growing interest and lessons learned from outside organisations, the threat to patient safety remained, as evidenced by the events at Mid Staffordshire NHS Foundation Trust, where an estimated 400 to 1200 patients died due to a combination of staffing shortages, financial pressures and a poor organisational culture which led to the tolerance of inferior practice (Ball et al., 2013; Francis, 2013). Today, as the NHS faces unprecedented operational and financial pressures, while recovering from the ongoing pandemic, it is clear that patient safety is under significant threat.

While research and interest concerning patient safety has grown substantially in hospitals and primary care, one healthcare setting which is routinely overlooked is the ambulance and emergency services, where the risk to patients is significant (Bigham et al., 2011, 2012; Fisher et al., 2015). When comparing the ambulance and emergency services with other care settings, it is clear that they have been understudied and neglected in the literature, and although the interest has risen slightly in the previous few years, a large gap remains (Bigham et al., 2011, 2012; Boysen et al., 2019; Ebben et al., 2017; Fisher et al., 2015; Hofoss & Deilkås, 2008; Illingworth, 2015). Exploring perceptions of patient safety by staff in the NHS ambulance services is therefore necessary to provide a deeper understanding of their concerns and priorities, as well as highlight issues they view as important to address. This study aimed to capture these perceptions of patient safety across all organisational levels in three English ambulance service NHS trusts.

## Methods

A generic qualitative approach was utilised for this study due to its appropriateness for exploring the perceptions of individuals involved in a phenomenon, the exploratory nature of the research aim, as well as the congruence of its theoretical perspective and methods of data collection and analysis (Merriam, 2002). Using a methodological approach based upon the principles of generic qualitative inquiry, we conducted semi-structured interviews with staff representing three levels in the organisation – operational, managerial and executive – as well as across three ambulance service NHS trusts in England. The results from the narrative review informed the development of the interview schedule by KS, SS, PW and CK, which was used during interviews to provide prompts for discussion in line with the aim and objectives of the study (Hart, 2018). Each interview was conducted and transcribed by KS, and the data were analysed by KS under the supervision and guidance of SS, PW and CK, using the framework method of qualitative data analysis (Gale et al., 2013). The concept of saturation was considered; however, a more structured approach was adopted due to several practical and theoretical considerations of the sample and timescale of the study (Saunders et al., 2018). Instead of using trustworthiness, the authors borrowed similar concepts from constructivist and positivist paradigms to measure, assess and establish a high standard of quality of this qualitative study through reliability, transferability and validity (Koch, 2006; Lincoln & Guba, 1985; Morse et al., 2002).

### Sampling

Given the large number of participants required, the gatekeeper in each NHS trust, often a research paramedic, adopted a joint purposive and snowball sampling method to aid in the recruitment of 44 participants representing three distinct ambulance service NHS trusts and three organisational levels. Snowball sampling helped to ensure that the recruitment targets were achieved, especially when gatekeepers did not have the time to find interested staff. Participants were categorised as operational, managerial or executive staff. Table 1 demonstrates the types of roles sought and their respective categories, in which three to eight participants were recruited within each organisational level in the three NHS trusts (Table 2).

**Table 1.** Organisational categorisation of staff.

Executive-level staff	Management-level staff	Operational-level staff
Chief executive Deputy/assistant chief executive Director of performance of patient experience Director of service delivery Director of finance Director of organisational development Director of workforce development Head of education, training and development Medical director Assistant director of healthcare governance	Head of clinical safety Assistant clinical director Consultant paramedic Advanced/specialist paramedics Head of risk and safety Risk manager Head of clinical governance Head of clinical education	EOC staff Paramedic

EOC = emergency operations centre.

**Table 2.** Total number of participants by NHS trust and organisational level.

	Trust T1	Trust T2	Trust T3	Total
**Executive**	4	3	3	10
**Management**	6	5	5	16
**Operational**	5	6	7	18
**Total**	15	14	15	44

**Table 3.** Sample demographic characteristics.

	Executive	Management	Operational
**Number per trust, N (%)**	T1: 4 (27%) T2: 4 (29%) T3: 3 (20%)	T1: 5 (33%) T2: 4 (29%) T3: 5 (33%)	T1: 6 (40%) T2: 6 (42%) T3: 7 (47%)
**Age, mean (min, max)**	T1: 52 (46, 56) T2: 54 (47, 66) T3: 49 (38, 57)	T1: 46 (43, 53) T2: 42 (29, 52) T3: 45 (31, 57)	T1: 39 (29, 49) T2: 36 (25, 45) T3: 30 (24, 42)
**Service duration in years, mean (min, max)**	T1: 24 (11, 31) T2: 7 (2, 11) T3: 13 (1, 27)	T1: 18 (0.5, 25) T2: 22 (11, 35) T3: 16 (5, 30)	T1: 15 (5, 27) T2: 10 (5, 14) T3: 6 (0.4, 16)
**Sex, N (%)**	Male: 7 (64%) Female: 4 (36%)	Male: 7 (50%) Female: 7 (50%)	Male: 12 (63%) Female: 7 (37%)
**Nationality, N (%)**	UK: 11 (100%) Overseas: 0 (0%)	UK: 14 (100%) Overseas: 0 (0%)	UK: 16 (84%) Overseas: 3 (16%)

## Results

The sample demographic characteristics are summarised in Table 3 to provide deeper insight into the age, service duration, sex and nationality of the 44 participants across the three organisational levels.

In total, 44 interviews were conducted from September 2017 to August 2018 (21 interviews in person, and 23 by telephone), lasting an average of 47 minutes. Five overarching dominant themes emerged from the analysis of the data, including the varied interpretation of patient safety, significant patient safety risks, reporting culture shift, communication and organisational culture.

### Varied interpretation of patient safety

During the interviews, participants often described their interpretation of patient safety, where it was clear that the responses were role and context dependent, as they varied according to their organisational level. Executive and management-level participants almost unanimously defined patient safety with a systems thinking approach; they described it as an existing feature permeating every aspect of the ambulance services and consisting of organisational-wide factors, which had an overarching influence on patient safety.

Patient safety is about making sure that everything that I put in place, my whole strategy that I put in place, my action plan that I put in place, has the patient at the very centre of it. [B5-T3]

Operational participants, on the other hand, defined their interpretation of patient safety as relating to preventing specific instances of patient harm, rather than involving any wider system and organisational factors.

that’s what defines patient safety, it’s the prevention of unnecessary harm and reduction of errors while treating patients. [C3-T2]

### Significant patient safety risks

Participants reported several significant risks which presented threats to patient safety, including service demand pressures, triaging, as well as a lack of training and deskilling. A large majority of participants were concerned about the increasing service demand pressures facing the NHS ambulance services and the negative impact these have on patient safety due to depletion of the workforce and infrastructural resources, the influence on human factors, as well as the ultimate result of delayed care for patients.

As far as demand goes, people are more tired, so mistakes will happen. We’re doing 12-hour nights with no time in between jobs, and it naturally leads to a higher risk for patients. [C5-T2]

Beyond the patient safety risks presented by service demand pressures, participants also described how triaging, or prioritising and allocating care for patients due to the inadequacies of the NHS Pathways system, and the lack of clinical knowledge held by staff in the emergency operations centre had a substantial negative impact on patient safety.

the categorisation system which categorises calls … leaves like elderly fallers as a low category, so it leaves vulnerable people on the floor for quite a long time. [C3-T3]

Lastly, participants suggested that another significant risk to patient safety was the lack of training offered to front-line staff, and how they were then more at risk of deskilling, or losing their clinical knowledge and skillsets, thereby endangering patients.

there’s an element of deskilling going on and those staff going out probably aren’t as well trained as they should be … so the care that’s going to come out of that is probably not going to be as safe or as consistent as I would like it. [C1-T1]

### Reporting culture shift

Participants overwhelmingly perceived the reporting culture in the past NHS ambulance services as having been poor, where front-line staff were fearful of reporting patient safety incidents (PSIs) due to the existence of a pervasive blame culture, contributing to an expectation and fear of punitive measures for reporting incidents.

If you made a clinical mistake, you wouldn’t speak about it for fear of being punished. [A3-T1]

Participants suggested that even if there had not been an evident blame culture in the NHS ambulance services, they would not have reported incidents anyway, as the organisation did not encourage reporting issues, nor did it have a robust reporting system or infrastructure in place.

thirty years ago, you wouldn’t have had any reporting system or step-by-step process to follow. [B1-T2]

While all participants emphasised the negative reporting culture of the past, they also expressed that the reporting culture in the NHS ambulance services had since improved and has largely eliminated the blame culture by transitioning to one which is open and where staff feel supported by their organisations to report incidents. Participants also described how the NHS ambulance services now had a robust reporting system, Datix®, which made reporting incidents easier than it had been in the past.

I do think it is a lot better; I’ve seen like a massive change, and like I’ve seen people reporting things that … in the past would have gone unreported. [C3-T3]

### Communication

A majority of participants perceived that the communication between staff had a significant impact on patient safety in the NHS ambulance services and raised several communication issues presented by workforce and infrastructural resources. Infrastructural resources, or the physical and organisational structures in place, included the inconsistent and ineffective use of multiple channels of communication, where staff would use their favourite means of communication while ignoring the others. For instance, participants discussed how some staff would only rely on communication received over emails, while ignoring all other channels of communication and missing vital information, thereby having a substantially negative impact on the safety of patients.

publishing it on paper doesn’t hit everybody, publishing it electronically doesn’t hit everybody. So it’s a bit of a stumbling block about getting the information out about what’s safe and what’s unsafe, what works and what doesn’t work. [B2-T2]

In addition to the inadequate use of the channels of communication, participants also referenced how the IT infrastructure in the NHS ambulance services was largely antiquated and not fit for purpose, both of which impact patient safety negatively.

we are digitally immature, we don’t have a really good infrastructure to utilise modern technology. [A3-T1]

Participants also raised how workforce resources, such as the operational pressures and the geographically dispersed and mobile nature of their work, presented barriers to communication.

clinicians don’t get to sit down in a mess room together, and they never get to see each other from one start of their shift to the end, so there’s no real opportunity for people to bounce problems or concerns off each other. [B3-T2]

### Organisational culture

Almost all 44 participants emphasised that organisational culture had a substantial impact on patient safety in the NHS ambulance services, where they focused on the historical remnants of the organisational and cultural legacy, which continued to have a negative impact on patient safety.

it’s the entrenched, embedded culture that’s been difficult to change. [A1-T2]

A majority of participants discussed the focus on time targets by higher level staff, as well as the aversion to risk and a command-and-control hierarchical structure, which had remained relatively unchanged from the past.

that target of that delivery of that seven minutes, eight, 19 minutes, half an hour, 40 minutes for all those categories, is putting a pressure on everything that stops some of the safety things happening. [B5-T2]

it’s very much a command and control, so it’s a very hierarchical type of environment that you work in. [B5-T1]

In addition, participants described how the historical remnants could be addressed, to have a positive impact on the organisational culture and patient safety, by becoming a learning organisation. According to participants, this would involve embedding a culture of learning through continually training and educating all levels of staff, flattening any existing hierarchical elements and implementing leadership at all levels to facilitate these changes.

that sort of hierarchy, which needs to be flattened … and I’m reducing the number of senior people within my team to make a flatter structure, and I think that will just open the door a bit more. [A2-T3]

The organisational culture and leadership needs to make sure that the ethos of patient safety and ethos of doing the right thing for the right patient at the right time. [B3-T3]

## Discussion

### Varied interpretation of patient safety

This study is the first of its kind to highlight an in-depth understanding of how staff from all organisational levels interpret patient safety, including how it is role and context dependent for staff. It was evident that management and executive-level staff interpreted it using a holistic and comprehensive systems thinking approach, while operational staff understood it as only relating to the treatment of patients, in line with the scope of their roles and responsibilities. The existing literature has demonstrated a lack of a standardised classification of patient safety, as the definition, measurement and interpretation of the concept varies widely across healthcare and research. This inconsistent approach arguably creates a barrier to the advancement of patient safety strategies given the absence of a uniform understanding of patient safety and its related concepts (Emanuel et al., 2008; Fisher et al., 2015; Kim et al., 2015; Sherman et al., 2009; World Health Organization, 2017).

### Significant patient safety risks

According to participants, the most significant risks to patient safety in the NHS ambulance services included service demand pressures, triaging, the deskilling of clinical staff and lack of training. In particular, participants perceived that the risks to patient safety presented by the increasing demand in the NHS ambulance services by service users were momentous and were emphasised throughout the interviews as a result. Although it was clear that some risks to patient safety were only known to certain subgroups of staff, all participants representing the three organisational levels were exposed to the substantial pressure to respond to the rise in demand. Somewhat surprisingly, there is not a large evidence base focusing on the risks presented to the NHS ambulance services by the rising demand. While the topic of increasing demand is present in the literature, it is largely disregarded as a standalone patient safety risk and is instead described as an underlying factor amplifying the risk presented by other issues.

Beyond demand, participants also emphasised the infrastructural and workforce resources involved in triaging patient care and how they constituted a significant risk to patient safety in the NHS ambulance services. There is an absence of evidence which has explored the processes involved in triaging and the patient safety risks they present, potentially representing a reflection of the wider lack of literature around patient safety in this care setting (Huibers et al., 2011; Lidal et al., 2013; Turner et al., 2008, 2017). While the literature is lacking in this area, one study by Fisher et al. (2015) found that triaging and call-handling were rated as the third highest patient safety risk by medical directors in the NHS ambulance services, thereby supporting this finding. Lastly, the perception of participants that there was a severe lack of training available to staff in the NHS ambulance services, which resulted in the subsequent deskilling of clinical staff, was supported by the broader literature (Atack & Maher, 2010; Bigham et al., 2011; Chesters et al., 2016; Fisher et al., 2015; O’Cathain et al., 2018; O’Hara et al., 2015).

### Reporting culture shift

The available evidence in the literature has identified the widespread existence of a poor reporting culture across the ambulance and emergency services, where front-line staff are less inclined to report errors and mistakes due to an expectation of retribution or punishment, ultimately resulting in a high rate of unreported PSIs (Bigham et al., 2011, 2012; Chesters et al., 2016; Fairbanks et al., 2008; Fisher et al., 2015; Ingram et al., 2019; Kirk et al., 2018; Morello et al., 2012; O’Hara et al., 2014, 2015; Sinclair et al., 2018; Verbakel et al., 2015). Within the United Kingdom, Kirk et al. (2018) recently found that there is a lack of evidence which indicates that the NHS ambulance services have transitioned away from a culture of blame to one that is more open where staff feel comfortable to raise concerns or issues. Therefore, this study is arguably the first to provide evidence of a shift away from an extensive blame culture to one where front-line staff now feel supported and free to report incidents without fear of punishment or bullying from their superiors. Participants reported that due to NHS trust-wide initiatives, as well as the introduction of the electronic reporting system, Datix®, the perceptions of reporting culture had significantly improved within the previous couple of years, and that as a result, the number of incidents being reported had risen considerably.

### Communication

This study arguably provides some of the first evidence of the perceptions of staff in the NHS ambulance services concerning the relationship between communication and patient safety, including barriers to effective communication, as well as solutions and its impact on the safety of patients. Almost all participants discussed the relationship between patient safety and communication and how poor communication was detrimental to patient safety, while robust communication led to high-quality patient care. In particular, participants referenced how the infrastructural and workforce resources in the NHS ambulance services engendered four major communication issues, including the mobile and dispersed workforce, the ill-equipped and outdated IT infrastructure, as well as the ineffective use of multiple communication channels and significant operational pressures. There is an absence of historical literature that explores this relationship, thereby causing issues with comparing these findings with the available evidence. Systematic and scoping reviews by Bigham et al. (2012) and Fisher et al. (2015), respectively, found that there has been minimal research conducted which focused on communication in the ambulance and emergency services, as well as that a majority of research emphasised inter-communication between different care settings during the handover of patient care, instead of intra-communication between staff in the NHS ambulance services, which this study explored.

### Organisational culture

Participants from all three organisational levels perceived that the organisational culture had a sizeable impact on patient safety. In particular, participants also highlighted the existence of remaining and detrimental historical remnants which were left over from an organisational and cultural legacy in the NHS ambulance services. According to participants, these remnants included an emphasis on time targets and staff who were overly risk-averse, as well as a staffing structure that was hierarchical in nature. In addition, participants described a recent shift in the organisational culture, which they perceived to be positive. It was also noted that the NHS ambulance services were becoming a learning organisation by providing staff with ongoing training and education, flattening the hierarchical structure of staff, as well as by empowering staff to develop leadership skills in every role across the organisation. This finding that patient safety and organisational culture were interconnected was supported by the literature, which has found that in addition to altering any procedural and structural aspects, the organisational culture must also be improved to result in an increased level of safety for patients (Braithwaite et al., 2017; Curry et al., 2017; Kaufman & McCaughan, 2013; Knowles et al., 2018).

## Conclusion

The results from this study highlight the belief of participants that the NHS ambulance services are becoming safer for patients, thereby providing some indication of an awareness of the historical patient safety issues and the ways that they are being addressed. Most notably, the perception of participants that the reporting culture in the NHS ambulance services had been improved significantly in the past few years, which has not been yet demonstrated in other similar research, was of particular importance. The involvement of 44 participants representing three organisational levels of staff and three ambulance service NHS trusts in England produced a deep understanding of the perceptions of patient safety in the NHS ambulance services. The consistency of their responses across the three organisational levels and NHS trusts indicates that the issues identified in the study may be generic and have application in other ambulance and emergency services, both in the United Kingdom and internationally.

### Limitations

While it is expected that the findings remain relevant today, the interviews were conducted from 2017 to 2018 and should be viewed with this in mind. Although 44 participants were interviewed across multiple organisational levels and NHS trusts, the sample size remains small; therefore, additional research is required to substantiate these qualitative findings, as well as to further explore topic areas highlighted in this study. While it also represented a strength of the research, a limitation of the study was the categorisation and allocation of participants into three organisational levels. Although a systematic approach was utilised, it is expected that there was some lack of consistency given that the roles and titles varied in the three NHS trusts. The five dominant themes described in the findings were said to represent the perceptions of patient safety of staff in the NHS ambulance services; however, these may be more reflective of the prompts used in the interview schedule and unrepresentative of all perceptions of patient safety. Although the qualitative methodology enabled the in-depth probing of topics which would have been infeasible using a quantitative survey, the unknown nature of whether certain perceptions were not captured represents a limitation (Barriball & While, 1994; Percy et al., 2015). Despite the fact that perceptions were captured from participants representing several NHS trusts and organisational levels, the findings are not representative of the 35,000 staff employed by the NHS ambulance services and cannot be broadly generalised.

The research was conducted by KS for the fulfilment of the requirements for a PhD. KS did not have a background in the NHS ambulance services; however, their neophyte nature was viewed as a strength as it facilitated a less biased approach when collecting and analysing the data given that no preconceptions, either from working or studying the NHS ambulance services previously, were present.

## Acknowledgements

The authors would like to thank all of the participating ambulance service NHS trusts in England and their kind staff who all gave their time in their busy schedules to contribute to this study. KS would like to thank Edge Hill University for funding the PhD Studentship and for providing the resources which made it possible to carry out this study.

## Author contributions

SS and PW conceived the study and all authors contributed to its design. KS carried out all of the data collection and analysis, while SS, CK and PW extensively supervised and provided support during the process. KS drafted the manuscript, and all of the authors reviewed and agreed on the current version. KS acts as the guarantor for this article.

## Conflict of interest

None declared.

## Ethics

This study first gained ethical approval from the Faculty of Health and Social Care Research Ethics Committee (FREC) at Edge Hill University (Ormskirk, England), before it then obtained approval from the Health Research Authority (HRA).

## Funding

This research received no specific grant from any funding agency in the public, commercial or not-for-profit sectors, and was instead funded by Edge Hill University as part of a PhD Studentship.
